# Nicotine and neuronal nicotinic acetylcholine receptors: unraveling the mechanisms of nicotine addiction

**DOI:** 10.3389/fnins.2025.1670883

**Published:** 2025-10-17

**Authors:** Jian Jiang, Xia Li, An-fu Hu, Guo-jun Zhou, Yi-han Gao, Chengyun Xu, Xi-mei Wu, Hong-Juan Wang

**Affiliations:** ^1^China Tobacco Zhejiang Industrial Co., Ltd., Hangzhou, China; ^2^Shanghai New Tobacco Product Research Institute Co., Ltd., Shanghai, China; ^3^Department of Pharmacology, Hangzhou City University School of Medicine, Hangzhou, China; ^4^Department of Pharmacology, Zhejiang University School of Medicine, Hangzhou, China; ^5^China National Tobacco Quality Supervision and Test Center, Zhengzhou, China

**Keywords:** nicotine, nAChRs, VTA-NAc, MHb-IPN, addiction

## Abstract

Nicotine, recognized as the principal addictive component in tobacco, is mechanistically linked to its interaction with neuronal nicotinic acetylcholine receptors (nAChRs). nAChRs are ligand-gated ion channels composed of five transmembrane subunits, with the α_4_β_2_ receptor subtype being the most common in the brain, playing a crucial role in the behavioral effects of nicotine. When nicotine binds to α_4_β_2_ nAChR, it significantly enhances the firing rate and burst firing of dopamine neurons in the brain, thereby activating the mesolimbic dopamine system. This system promotes the formation of nicotine addiction in the early stages of addiction through rewarding sensory stimulation and associative learning. The α_4_β_2_ nAChR subunit has been identified as the principal subtype implicated in the pathogenesis of nicotine addiction. However, other nAChRs subtypes also play important roles in the onset and maintenance of nicotine addiction. Understanding the relationship between nicotine addiction and nAChR subtypes is crucial for fully uncovering the neurobiological mechanism behind its addictive properties and lays the foundation for developing more targeted smoking cessation strategies.

## Highlights

- This review delineates the subtype-specific roles of nAChRs—such as α_4_β_2_, α_6_β_2_*, and α_5_-containing subtypes—in mediating nicotine rewards and aversion via distinct neural circuits.
- The VTA-NAc pathway is recognized for its dopaminergic mechanisms underlying rewards, whereas the MHb-IPN circuit is implicated in nicotine aversion through glutamatergic and GABAergic signaling.
- Genetic variants like CHRNA5 rs16969968 and stoichiometric differences among nAChR subtypes are identified as critical determinants of individual susceptibility to nicotine dependence.
- Integrating multi-system neurotransmitter interactions—including dopamine, glutamate, GABA, and GLP-1—offers a more comprehensive model of nicotine addiction that extends beyond traditional rewards pathways.

## 1 Introduction

Nicotine is the primary active component in tobacco products that causes addiction. Nicotine exerts both strong rewards and aversive effects in the central nervous system through its interaction with nicotinic acetylcholine receptors (nAChRs) (Maskos et al., 2005; Picciotto et al., 1998; Tolu et al., 2013). This process not only endows nicotine with high pharmacological activity in addiction but also complicates the mechanisms of its physiological and pathological effects resulting from direct or indirect activation of multiple intracellular signaling pathways. Nicotinic acetylcholine receptors are ligand-gated ion channels composed of five subunits, each containing an extracellular ligand-binding domain and four transmembrane regions (Le Novère et al., 2002; Lindstrom, 1997). In mammals, nAChRs subtypes are highly diverse, such as the most common α_4_β_2_ heteropentamer and α_7_ homopentamer in the brain (Maskos et al., 2005; Wooltorton et al., 2003). A comprehensive understanding of nAChR function is essential for analyzing the mechanisms of nicotine addiction. On one front, the elucidation of dynamic regulatory mechanisms governing nAChR structural plasticity and functional modulation promises to yield a more comprehensive understanding of molecular addiction processes. On another front, research into the distribution of nAChRs and their systemic impacts will help elucidate the broader physiological consequences of nicotine addiction. This review synthesizes current evidence on the relationship between nicotine addiction and nAChRs, and clarify the neurobiological basis of nicotine addiction.

## 2 Neurobiological basis of nicotine addiction

The rewarding mechanism of nicotine addiction exhibits complex biphasic regulatory characteristics, where the dynamic equilibrium between positive reinforcement (such as euphoria) and negative regulation (such as aversive reactions) constitutes the neurobiological basis for the formation of addictive behaviors. The rewarding effects of nicotine typically manifest as sensations of “lightheaded euphoria” or “excitement” post-consumption, while aversive effects are reflected in discomfort reactions such as nausea and dizziness (Corrigall et al., 1992; Koob, 1992; Villanueva et al., 1989). The balance of these effects is closely associated with individual-specific factors, including dosage, personal sensitivity, and tolerance development. The addictive properties of nicotine are predominantly mediated by the integration of interactive signaling processing rewards and aversion across multiple brain regions. As the central hub of the mesolimbic dopamine system, dopaminergic neurons in the ventral tegmental area (VTA) form critical neural circuits through their projections to the nucleus accumbens (NAc) and prefrontal cortex, constituting the neural substrate for nicotine’s rewarding effects (Clarke et al., 1988; Corrigall et al., 1994; Nisell et al., 1996).

### 2.1 Rewarding mechanisms in nicotine addiction

The core pathological mechanism of nicotine addiction involves the rewards modulation system of the mesolimbic pathway. This system generates positive reinforcement signals primarily through dopaminergic transmission within the mesolimbic circuit, mediated by dynamic interactions between the ventral tegmental area (VTA) and the nucleus accumbens (NAc).(Fu et al., 2003; Mansvelder and McGehee, 2000; Rice and Cragg, 2004; Schwartz et al., 1984). Research has suggests that nicotine produces rewarding effects not through a single neurotransmitter system, but through the integrated actions of dopaminergic, GABAergic, glutamatergic systems and atypical rewarding pathways, which together facilitate spatiotemporally specific neuroplastic adaptations (Wooltorton et al., 2003; Grieder et al., 2019; Mansvelder et al., 2002). This multidimensional regulatory mechanism explains how nicotine induces rapid addiction.

As a high-affinity agonist of nicotinic acetylcholine receptors (nAChRs), nicotine directly activates VTA dopamine neurons through β_2_ subunit-containing receptors, inducing Na^+^/Ca^2+^ influx that causes membrane depolarization. This enhances the firing frequency of dopaminergic neurons and triggers transient surges of dopamine release in the NAc. This process is completely abolished in β_2_ subunit knockout mice, confirming its role as the molecular basis of rewarding effects (Maskos et al., 2005; Picciotto et al., 1998; Tolu et al., 2013; Dani and Bertrand, 2007; De Biasi and Dani, 2011; Mao et al., 2011; Pidoplichko et al., 1997). Nicotine not only directly acts on dopaminergic neurons, but also transiently enhances GABAergic neurons’ inhibitory drive on dopaminergic neurons by binding to nAChRs within GABAergic neurons in the VTA (Wooltorton et al., 2003; Grieder et al., 2019; Mansvelder et al., 2002). In the initial stage, nicotine activates GABAergic interneurons through α_4_β_2_-nAChRs, increasing their spontaneous discharge frequency and thereby augmenting inhibitory inputs to dopaminergic neurons; However, with rapid receptor desensitization, the GABAergic inhibitory effects attenuate following sustained exposure, forming a “disinhibition-excitation potentiation” delayed reinforcement pattern. This temporal dissociation characteristic may underlie the dual-phase reinforcement properties of nicotine rewards. Due to the rapid desensitization of α_4_β_2_ nAChRs, when exposed to sustained low concentrations of nicotine, the GABAergic drive gradually diminishes over time, thereby relieving inhibition on dopaminergic neurons and ultimately enhancing their excitability. This phenomenon regulates the activity states of VTA dopamine neurons through dual mechanisms, playing a critical role in the process of nicotine-induced rewarding effects (Mansvelder et al., 2002; Yan et al., 2019). In the context of long-term effects, presynaptic α_7_nAChRs further promote the long-term excitability of dopaminergic neurons by enhancing glutamatergic inputs (Mao et al., 2011; Ostroumov and Dani, 2018; Pidoplichko et al., 2004). The combination of enhanced synaptic input and removal of inhibitory constraints constitutes a critical step in the initiation of nicotine addiction.

The nicotine rewarding effect is further complicated by its direct regulation of dopamine release within the striatum. In the nucleus accumbens core and dorsal striatum, dopamine release is regulated by presynaptically expressed heteromeric nAChRs, particularly those mediated by receptors containing α_6_, α_4_, β_2_, and β_3_ subunits (Salminen et al., 2004; Zoli et al., 2002). Nicotine activates these receptors to physically enhance dopamine release while concurrently reducing basal dopamine levels. Although this rapid desensitization phenomenon appears paradoxical to the rewarding mechanism, it effectively enhances the signal-to-noise ratio of dynamic rewarding signals by reducing background dopamine “noise,” thereby strengthening the coupling between nicotine rewarding and environmental cues (Rice and Cragg, 2004; Threlfell and Cragg, 2011; Zhang et al., 2009). This signal optimization mechanism makes nicotine a particularly substance prone to induce addiction.

Studies further reveal that nicotine’s rewarding effects are not limited to the regulation of the dopamine system. Nicotine can directly act on brain regions outside the mesolimbic dopamine system, such as the central linear nucleus and parabrachial nucleus, and manifests rewarding effects independent of dopamine through interactions with opioid receptors or other neuropeptides, suggesting a multi-system interactive rewarding integration mechanism (Ikemoto et al., 2006; Neugebauer et al., 2011; Trigo et al., 2009).

In summary, the core of the nicotine rewarding mechanism lies in its profound impact on dopaminergic transmission in the mesolimbic system, achieved through complex direct and indirect pathways. Starting with high-affinity β_2_ subunit-containing nAChRs, nicotine directly activates VTA dopamine neurons; concurrently, it enhances phasic dopaminergic signaling and optimizes the signal-to-noise ratio of rewarding signals by modulating GABAergic and glutamatergic inputs. These synergistic neuroregulatory mechanisms collectively establish nicotine as a substance with potent reinforcing properties, cementing its central role in addictive behaviors.

### 2.2 Aversive effects of nicotine addiction

The formation and maintenance of nicotine addiction fundamentally constitutes a neurobiological process involving dynamic interactions between rewarding and punishment mechanisms. Within this framework, punishment mechanisms exert critical constraining effects on nicotine-seeking behavior through negative reinforcement effects mediated by specific neural circuits. Recent studies have revealed that the medial habenula (MHb)-interpeduncular nucleus (IPN) pathway serves as the central hub mediating nicotine’s aversive effects (Fowler and Kenny, 2011; Jensen et al., 2015; Sartor et al., 2010).

Through optogenetics, chemogenetics, and molecular imaging techniques, researchers have systematically elucidated the aversion signaling pathway mediated by α_3_/α_5_/β_4_ nicotinic acetylcholine receptors (nAChRs) within this neural circuit (Girod et al., 2000; Grady et al., 2001, 2009; Ren et al., 2011). The high-affinity binding of nicotine to α_5_ subunit-containing receptors on MHb neurons triggers Ca^2+^-dependent burst firing, which induces the axonal terminal release of glucagon-like peptide-1 (GLP-1). This subsequently activates GLP-1 receptors in the IPN to promote cAMP production, significantly enhancing the excitability of glutamatergic neurons in the IPN (Hussain et al., 2008; Sherafat et al., 2020; Tuesta et al., 2017). Studies on gene knockout mice have demonstrated that deletion of the α_5_ nAChR subunit significantly reduces nicotine’s aversive effects, enabling animals to tolerate higher doses of nicotine (Fowler et al., 2011). The missense mutation rs16969968 in the CHRNA5 gene results in the substitution of aspartic acid with asparagine at position 398 of the encoded α_5_ subunit. This variant exhibits decreased sensitivity to nicotine agonists and reduced calcium permeability, substantially elevating the risk of nicotine addiction (Bierut, 2010; Breetvelt et al., 2012; Buczkowski et al., 2015; Liu et al., 2010).

The IPN acts as a relay station for aversive signals, regulating downstream neural activity through dual projection pathways: its GABAergic fibers directly inhibit cholinergic neurons in the laterodorsal tegmental nucleus (LDTg), while glutamatergic projections activate an NMDA receptor-dependent negative regulatory network within the VTA (Ables et al., 2017; Dautan et al., 2016; Lima et al., 2017; Liu et al., 2022; Quina et al., 2017; Wolfman et al., 2018). Optogenetics experiments demonstrate that specific activation of the IPN→LDTg GABAergic pathway induces robust place avoidance behavior, while inhibition of this pathway completely abolishes the aversive effects of high-dose nicotine (Wolfman et al., 2018; Alderson et al., 2005; Ishibashi et al., 2009; Maskos, 2008). The LDTg reduces glutamatergic input strength to VTA dopamine neurons through GABAB receptor-mediated presynaptic inhibition mechanisms, establishing functional antagonism against the rewarding system (Melani et al., 2019). This bidirectional regulation manifests behaviorally as dose-dependent biphasic effects: low-dose nicotine induces reward via VTA dopaminergic activation, while high-dose nicotine elicits aversion through the MHb-IPN-LDTg pathway, with the critical dose threshold being regulated by α_5_ subunit expression levels.

Besides regions like the VTA and the MHb-IPN pathway, the process of nicotine addiction also involves a range of other brain areas and related neurotransmitter systems. For example, the insular cortex is a key region in regulating nicotine intake and seeking behavior. Damage to this area can significantly reduce an individual’s craving for nicotine, making it a potential target for withdrawal and relapse interventions (Naqvi et al., 2007). Other studies have shown that certain cortical areas of brain, such as the prefrontal cortex and basolateral amygdala, are essential in strengthening the memory and relapse in the addiction process by integrating rewarding and emotional information (Forget et al., 2010; Kodas et al., 2007; Le Foll et al., 2008). Thus, nicotine is not merely a rewards-promoting substance that simply activates nAChRs, but rather a complex modulator capable of dynamically regulating dopamine, glutamate, GABA, and other neurotransmitters.

**FIGURE 1:**
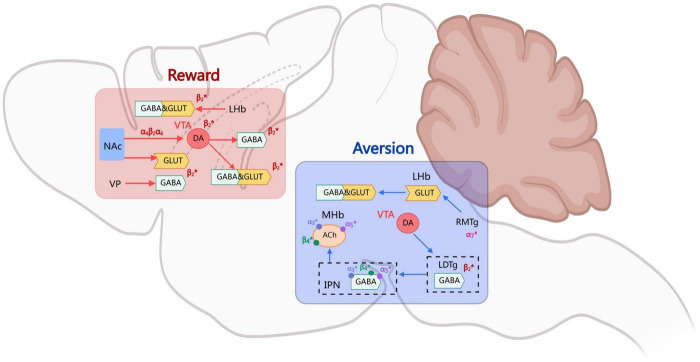
The mechanisms of nicotine rewards and aversion.

Overall, the integration of rewarding and aversive signals in the process of nicotine addiction depends on the dynamic interactions of multiple brain regions and neural circuits. The VTA-NAc pathway dominates the rewarding mechanism, while the MHb-IPN pathway regulates the aversive effect. At the same time, other regions of the brain, such as the insular cortex, also play a significant role in nicotine addiction and withdrawal (Figure 1).

## 3 Core mechanistic roles of nAChRs in nicotine addiction

**FIGURE 2:**
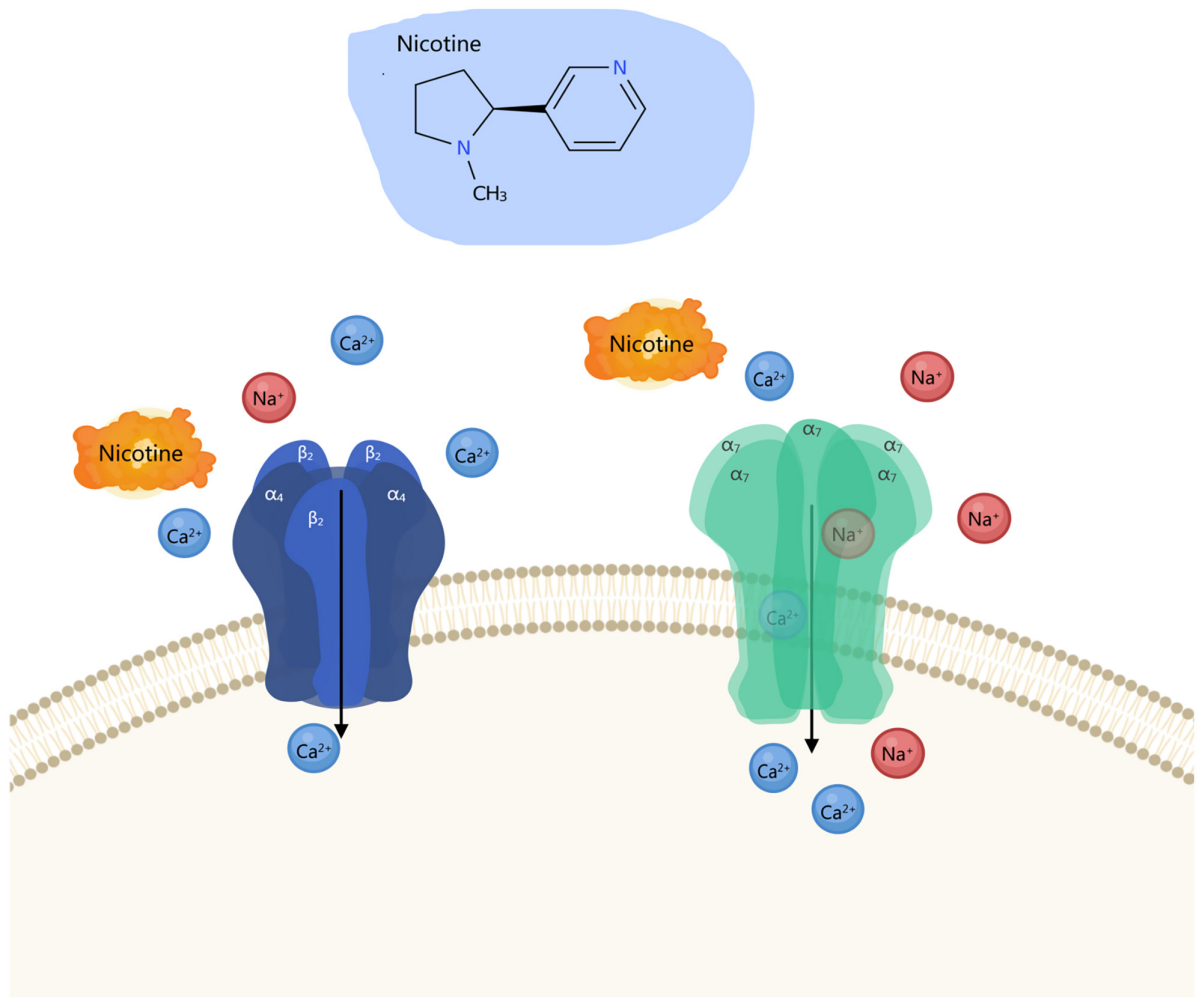
Schematic representation of nicotine’s effects.

As pivotal members of the ligand-gated cation channel superfamily, nicotinic acetylcholine receptors (nAChRs) serve as the cornerstone of nicotine addiction neurobiology, with the complexity of their molecular architecture and functional modulation forming its fundamental basis (Le Novère et al., 2002; Lindstrom, 1997). Since the groundbreaking discovery of acetylcholine as a neurotransmitter by Dale (1935), our understanding of nAChRs has evolved from a simple neuromuscular junction signaling apparatus to a molecular nexus mediating cross-system neural plasticity (Changeux, 2020; Hulme et al., 1990). nAChRs are composed of five subunits (α and β subunits) assembled into a pentameric structure, forming a central water-filled ion channel. To date, nine α subunits (α_2_–α_10_) and 3 β subunits (β_2_–β_4_) have been identified. These subunits combine in various configurations to form functionally diverse receptor subtypes, with the α_4_β_2_ and α_7_ subtypes being the most representative in the central nervous system (CNS). As an exogenous agonist, nicotine binds with high affinity to the ligand-binding site within the extracellular domain of receptors, triggering conformational changes that open ion channels. This facilitates transmembrane flow of Na^+^, K^+^, and Ca^2+^, inducing cell membrane depolarization and activating downstream signaling cascades (Figure 2) (Changeux, 2018; McKay et al., 2007). nAChRs are ubiquitously distributed across virtually all anatomical brain regions, including presynaptic and postsynaptic membranes, axonal terminals, and somatic compartments. Within the brain, nAChRs demonstrate remarkable heterogeneity, with distinct subtypes executing specialized functional roles in specific brain regions. For instance, α_4_β_2_ receptors are widely distributed in the VTA, NAc, and prefrontal cortex, playing a central role in regulating dopamine release and reinforcement learning (Maskos et al., 2005; Picciotto et al., 1998). In contrast, α_7_ receptors are primarily localized in dopaminergic neurons and participate in modulating long-term potentiation of glutamatergic neurons (Wooltorton et al., 2003; Mansvelder et al., 2002). Within the VTA, dopaminergic neurons exhibit highly heterogeneous expression of nAChR subtypes, predominantly α_4_β_2_ and α_6_β_2_β_3_ complexes. Activation of these receptors enhances burst firing in dopaminergic neurons, increases dopamine release in the nucleus accumbens, thereby forming rewarding signals for external stimuli such as nicotine (Lindstrom, 1997; Exley et al., 2011). On the other hand, in the MHb, the α_5_, α_3_, and β_4_ subtypes similarly play negative regulatory roles in nicotine uptake and aversive effects (De Biasi and Salas, 2008; Sheffield et al., 2000). This bidirectional regulatory mechanism reflects the integrative role of nAChRs within complex interregional brain networks.

The rewarding mechanism of nicotine addiction is closely linked to the subtype-specific functions and molecular diversity of nicotinic acetylcholine receptors (nAChRs), the core of which lies in the spatiotemporal regulation of neurotransmitter release by distinct nAChR subtypes within the mesolimbic dopamine system. As the most abundant subtype in the central nervous system, the stoichiometric ratio differences between (α_4_β_2_)_2_β_2_ and (α_4_β_2_)_2_α_4_ of α_4_β_2_ nAChR determine receptor sensitivity and functional characteristics toward nicotine: The (α_4_β_2_)_2_β_2_ subtype exhibits high agonist affinity, whereas the (α_4_β_2_)_2_α_4_ subtype demonstrates 3–4-fold enhanced activation efficacy despite lower affinity, establishing a dual regulatory paradigm of “high-affinity” and “high-efficacy” (Gotti et al., 2009). Gene knockout experiments confirm that deletion of α_4_ or β_2_ subunits completely blocks nicotine-induced burst firing of VTA dopamine neurons and dopamine release in the nucleus accumbens, while mice expressing hypersensitive α_4_ mutants exhibit exaggerated rewarding responses to low-dose nicotine (Maskos et al., 2005; Picciotto et al., 1998; Mameli-Engvall et al., 2006; McGranahan et al., 2011; Naudé et al., 2016; Peng et al., 2017; Tapper et al., 2004). Pharmacological studies further reveal that the α_4_β_2_* nAChR partial agonist varenicline significantly reduces nicotine self-administration by competitively inhibiting nicotine binding and attenuating dopamine release. Concurrently, dihydro-β-erythroidine (DHβE), a selective antagonist of β_2_ nAChR, also inhibits nicotine addiction (Coe et al., 2005; Ivanová and Greenshaw, 1997; Reperant et al., 2010). The upregulation of α_4_β_2_ receptor expression in VTA GABAergic neurons shows high correlation with nicotine addiction susceptibility. Positron emission tomography (PET) studies demonstrate a positive correlation between α_4_β_2_ receptor density and withdrawal difficulty in smokers, indicating its central role in the dynamic regulation of addiction progression (Brody et al., 2004). The functional differentiation of α_6_β_2_* nAChR subtypes within the mesolimbic system further enriches the complexity of nicotine rewarding mechanisms. The α_6_ subunit exhibits specific expression in VTA dopamine neurons and their striatal terminals, co-assembling with the β_3_ subunit to form high calcium permeability complexes: (α_6_β_2_)_2_β_3_ and (α_4_β_2_)(α_6_β_2_)β_3_ (Salminen et al., 2004; Zoli et al., 2002). These receptors demonstrate significantly higher nicotine sensitivity compared to other subtypes. Notably, α_6_ knockout mice exhibit abolished motivation for nicotine intake in both acute nicotine self-administration and two-bottle choice paradigms (Bagdas et al., 2019; Liu et al., 2012). The β_3_ subunit serves as an auxiliary component of α_6_-containing receptors, enhancing nicotine’s regulation of striatal dopamine release by promoting receptor maturation and membrane localization (Gotti et al., 2009; Moen et al., 2021). Knockout of the β_3_ subunit reduces α_6_ receptor expression in the striatum and attenuates nicotine-induced dopamine release, while allelic variations in the CHRNB3 gene cluster show significant association with nicotine addiction risk (Bierut et al., 2007; Gotti et al., 2005; Thorgeirsson et al., 2010; Wen et al., 2016). The α_5_ subunit incorporates into α_4_β_2_ receptors as a non-ligand-binding auxiliary subunit, forming (α_4_β_2_)_2_α_5_ complexes that exhibit enhanced calcium permeability compared to classical α_4_β_2_ receptors and demonstrate resistance to agonist-induced desensitization (Chatterjee et al., 2013). The loss-of-function mutation (D398N) at the CHRNA5 gene rs16969968 locus increases nicotine addiction risk by reducing receptor calcium permeability, while VTA dopamine neurons in α_5_-knockout mice exhibit attenuated responsiveness to nicotine (Kuryatov et al., 2008; Morel et al., 2014; Sciaccaluga et al., 2015; Tapia et al., 2007). The α_5_ receptor primarily regulates dopamine release in the dorsal striatum, demonstrating spatial differentiation from α_6_* receptor function in the nucleus accumbens. This anatomical specificity may explain nicotine’s differential regulation of distinct behavioral paradigms (Exley et al., 2012). In contrast, α_7_ homomeric nAChRs play a relatively limited role in nicotine rewarding mechanisms. Although it mediates enhanced glutamatergic inputs to the VTA, α_7_ knockout mice exhibited no behavioral differences in nicotine self-administration and conditioned place preference tests. Only female individuals showed reduced nicotine intake during chronic oral administration (Bagdas et al., 2019; Pons et al., 2008). While methyllycaconitine (MLA) demonstrates efficacy in attenuating nicotine self-administration behaviors, its non-selective pharmacological actions coupled with null results observed in α_7_-nAChR knockout models collectively suggest a dissociation from canonical α_7_-mediated pathways in eliciting this behavioral modulation (Bryant et al., 2002; Markou and Paterson, 2001; Salas et al., 2007).

In conclusion, the molecular structure, diverse combinations, widespread distribution, and functional variety of nAChRs collectively underpin their central role in nicotine addiction (Table 1).

**TABLE 1:** Major nicotinic acetylcholine recepto (nAChR) subtypes and their roles in nicotine addiction.

nAChR subtype	Main brain regions	Primary role	Nicotine-related effects
α_4_β_2_	VTA, NAc, cortex, hippocampus	Core mediator of rewards	High-affinity binding; drives dopamine release
α_6_β_2_* (± β_3_)	VTA DA neurons, striatum	Enhances dopamine signaling	High sensitivity to nicotine; α_6_ or β_3_ KO abolishes nicotine intake motivation
α_5_	MHb–IPN, striatum	Modulates aversion and intake	CHRNA5 variants (rs16969968) increase dependence risk; regulates dose control
α_7_	Cortex, hippocampus, VTA	Cognition, plasticity, minor role in rewards	Low-affinity; enhances glutamatergic inputs; limited role in nicotine self-administration
α_3_β_4_	MHb–IPN	Mediates aversive effects	Contributes to withdrawal and aversion at higher nicotine doses

## 4 Discussion

This article provides a detailed overview of the mechanisms underlying nicotine addiction and the roles of various nicotinic acetylcholine receptor (nAChR) subtypes in this process. Nevertheless, the mechanisms of nicotine addiction remain only partially understood. As discussed above, most studies have concentrated on α_4_, α_5_, α_6_, α_7_, and β_2_ nAChR subunits (Braunscheidel et al., 2024; Gu et al., 2019; Huang et al., 2022, 2025; Jackson et al., 2017; Rigotti et al., 2023; Sakkiah et al., 2020; Yang et al., 2023), whereas others—such as α_3_ (Icick et al., 2020), which is densely expressed in the mHb—have received far less attention. Evidence indicates that allelic variations in the CHRNA3 gene, which encodes the α_3_ subunit, are associated with an elevated risk of nicotine addiction, although the precise mechanisms remain unclear (Elayouby et al., 2021). Therefore, future mechanistic studies should not only focus on well-studied subunits but also expand to include understudied nAChRs, thereby enabling a more comprehensive understanding of how nicotine induces addiction.

In addition, emerging technologies are reshaping our understanding of nicotine addiction. For example, omics approaches are playing an increasingly significant role in mechanistic studies. Several groups have applied single-nucleus transcriptomics (snRNA-seq) to ventral tegmental area (VTA) neurons and glial cells across three stages—pre-addiction, addiction, and post-addiction—yielding deeper insights into nicotine-induced changes (Fan et al., 2024). Looking ahead, single-cell ATAC-seq (Kimbrough et al., 2021; Jackson et al., 2024), spatial transcriptomics (Scott et al., 2024), proteomics (Lee et al., 2021), and metabolomics (Lian et al., 2024) are expected to further advance nicotine addiction research. These methods can unravel molecular mechanisms across multiple levels: epigenetic regulation (gene switching), spatial organization (regional and cellular interactions), protein function (receptors and signaling pathways), and metabolic states (energy balance and neurotransmission). Integrating multi-omics data will enable construction of a complete causal chain—from chromatin remodeling → gene transcription → protein function → metabolic alterations → behavioral phenotypes. This systems-level framework will provide valuable resources for identifying biomarkers and therapeutic targets, ultimately laying the groundwork for personalized smoking cessation strategies.

## 5 Conclusion

Nicotine addiction arises from the diversity and dynamic regulation of nAChR subtypes, which shape the balance between rewards and aversion in mesolimbic circuits. Recent evidence highlights the critical role of α_4_β_2_ and α_7_ receptors in modulating dopamine release through subtype-specific stoichiometry and calcium permeability, while α_5_-containing assemblies have been identified as genetic determinants of addiction vulnerability. At the same time, the MHb–IPN pathway, mediated by α_3_α_5_β_4_ receptors, has been increasingly recognized as a central hub for aversive modulation, expanding the traditional dopamine-centered framework. Technological advances in single-cell transcriptomics and spatial multi-omics now allow unprecedented resolution of subtype distribution and plasticity. This review argues that future research should integrate molecular, circuit, and behavioral perspectives, with emphasis on cell-type–specific receptor dynamics, adaptive plasticity under different nicotine exposure conditions, and individual genetic risk factors. Taken together, these insights suggest that nAChR subtype heterogeneity is not only fundamental to the mechanisms of nicotine addiction but also provides a foundation for precision strategies in smoking cessation.
